# Adiponectin Receptors Are Less Sensitive to Stress in a Transgenic Mouse Model of Alzheimer's Disease

**DOI:** 10.3389/fnins.2017.00199

**Published:** 2017-04-11

**Authors:** Zoltán P. Várhelyi, János Kálmán, Zita Oláh, Eszter V. Ivitz, Eszter K. Fodor, Miklós Sántha, Zsolt L. Datki, Magdolna Pákáski

**Affiliations:** ^1^Department of Psychiatry, Faculty of Medicine, University of SzegedSzeged, Hungary; ^2^Institute of Biochemistry, Biological Research Centre of the Hungarian Academy of SciencesSzeged, Hungary

**Keywords:** adiponectin, AdipoR1, AdipoR2, leptin, LepR, Alzheimer's disease, stress

## Abstract

**Background:** Adiponectin and leptin are implicated in the initiation and pathomechanism of Alzheimer's disease (AD). The serum concentrations of these adipokines has been extensively studied in AD, however little is known about their receptors in this disease.

**Objective:** We developed a novel approach to examine whether the receptors of adiponectin (AdipoR1 and -R2) and/or leptin (LepR) can contribute to AD pathomechanism. To achieve this, we investigated the effect of both genetic and environmental factors associated with AD on the expression of these receptors.

**Method:** We used C57BL/6J (WT) and APP(swe)/Presen(e9d)1 (AD) mice. Both strains were exposed to restraint stress (RS) daily for 6h over different time periods. Then, we measured the mRNA expression of AdipoR1, AdipoR2 and LepR and the level of AdipoR1 and AdipoR2 proteins in the hippocampal and prefrontal cortical areas of each mouse.

**Results:** We detected brain region specific transcriptomic changes of adiponectin receptors induced by APP and PS1 transgenes. Both acute and chronic RS caused significant elevations in AdipoR1 mRNA expression in the hippocampus of WT mice. In the prefrontal cortex, the mRNA expression of AdipoR1 followed a biphasic course. In AD mice, RS did not promote any changes in the expression of AdipoR1 mRNA and AdipoR1 protein levels. AdipoR2 mRNA in AD animals, however, showed a significant increase in the prefrontal cortex during RS. Regarding AdipoR1 and AdipoR2 mRNA and protein expression, relevant changes could be measured during stress exposure in both brain areas. Furthermore, stress exposed groups exhibited little change in LepR mRNA expression.

**Conclusion:** Our findings indicate that carrying the transgenes associated with AD induces modification in the expression of both adiponectin receptors. In the case of a normal genetic background, these receptors also appear to be sensitive to environmental factors, while in a genetically determined AD model less response to stress stimuli could be observed. The results suggest that modification of adipokine receptors could also be considered in the therapeutic approach to AD.

## Introduction

Alzheimer's disease (AD) is one of the most common forms of neurodegenerative dementia, being responsible for 60–80% of all recorded dementia cases (Alzheimer's Association, 2015). The pathogenesis of AD appears to be multifactorial in nature, with both genetic and environmental factors contributing to the development and progression of the disease. For example, obesity and one of its comorbidities, metabolic syndrome, are also associated with AD pathogenesis. Studies hint at the fact that members of the adipokine molecular group may be involved in the link between obesity and AD. Adiponectin and leptin are the most well characterized adipokines regarding their role in AD. These small peptides have two common traits: (1) They can be synthesized by the adipose tissue; (2) they have a specific cytokine and/or hormone function (Alzheimer's Association, 2015). Recently a model was proposed suggesting that decreased plasma adiponectin and increased plasma leptin concentrations, due to obesity in mid-life, can make the brain more susceptible to dementia, whereas weight loss in late-life and during the early periods of dementia leads to lower leptin and higher adiponectin levels and may have the same results, also contributing to the progression of neurodegeneration (Ishii and Iadecola, 2016).

The level of the 30 kDa adipocyte-derived polypeptide, adiponectin, is inversely associated with metabolic syndrome. Not only individual investigations (Une et al., 2011; Khemka et al., 2014), but also a meta-analysis, demonstrated the higher serum levels of adiponectin in AD patients (Ma et al., 2016). It has been found that an upregulation in adiponectin expression may be associated with mild cognitive impairment (MCI) and AD (Une et al., 2011). In addition, higher adiponectin levels were associated with smaller hippocampal volume, poorer performance in language, memory and global cognitive domains, and higher odds of MCI among women (Wennberg et al., 2016). Adiponectin has been proposed as a therapeutic target in certain cognitive deficiencies (Chan et al., 2012; Diniz et al., 2012). Adiponectin exerts its effects by binding to its two transmembrane receptors, adiponectin receptor 1 (AdipoR1) and adiponectin receptor 2 (AdipoR2), which are widely expressed in various areas of the brain, including the hippocampus (Qiu et al., 2011). Adiponectin enhances AMP-activated protein kinase (AMPK) activity via AdipoR1 to stimulate food intake and decrease energy consumption (Kadowaki et al., 2008). Also, AMPK activation represses amyloidogenesis, decreases mTOR signaling and enhances autophagy and lysosomal degradation of Aβ (Godoy et al., 2014). Adiponectin secretion is also upregulated by PPAR-γ induced activation of AdipoR2 (Kadowaki et al., 2008). The mechanistic link between PPAR-γ and amyloid clearance has been demonstrated to ameliorate the pathological and behavioral deficits in an AD mouse model (Mandrekar-Colucci et al., 2012). This is in contrast with the fact that both adiponectin receptors are involved in the regulation processes of amyloidogenesis. However, these receptors have not been studied in AD patients or in any animal models of the disease.

Leptin is a small, 16 kDa adipose-derived hormone which was found to be in lower concentrations in the blood of AD patients (Power et al., 2001). At the same time, the level of leptin in the cerebrospinal fluid (CSF) in a small cohort of patients with AD was significantly elevated (Bonda et al., 2014). Recently, another research group observed no difference in the CSF leptin levels of patients with AD or MCI (Maioli et al., 2015). One possible explanation for the paradoxical findings may lie in the signaling pathway of leptin in the central nervous system. Leptin receptor (LepR) activation in hippocampal neurons may play an important role in the memory of mice in relation to food intake and learning (Kanoski et al., 2011). By binding to LepR, leptin activates several intracellular signaling pathways, including signal transducer and activator of transcription 3 (STAT3), mitogen-activated protein kinase (MAPK) and phosphatidylinositol 3-kinase (PI3K) pathways to reduce the risk of AD (Li et al., 2016).

In addition to obesity, another possible environmental component in AD induction is psychological stress. Epidemiological studies indicate that stress is associated with increased risk of dementia and AD (Wilson et al., 2006). Repeated exposure of rats to mild stressors resulted in enhanced expression of adiponectin mRNA, suggesting that adipose tissue is sensitive to environmental stress (Sato et al., 2011). However, the effect of stress on the expression of adiponectin receptors has not yet been published in a scientific paper. Different types of acute stress, such as immobilization, forced swimming or noise increased the circulating levels of leptin (Haleem, 2014). Unlike acute stress, chronic unpredictable mild stress induced a decrease in serum leptin levels and in hypothalamic leptin receptor mRNA expression (Ge et al., 2013).

The primary aim of this study was to investigate the mRNA and protein expression of AdipoR1, AdipoR2 and leptin receptor (LepR) in an amyloid precursor protein (APP)/presenilin-1 (PS1) transgenic mouse model. Since obesity and stressful life events frequently co-occur, we also wanted to examine the effect of restraint stress (RS) on these adipokine receptors' expression in a quantifiable manner to find out whether stress, as a possible factor in the development of AD, may be able to influence AD pathology through these receptors. As AD affects the hippocampus and the prefrontal cortex most prominently, we selected these brain areas as the focal points of our experiment.

## Materials and methods

### Animals

Two separate mouse strains were used: a wild type C57BL/6J mouse strain (WT mice, *n* = 30) and an APP(swe)/PS1(e9d)1 transgenic mouse strain (AD mice, *n* = 30). Transgenic mice were purchased from the Jackson Laboratory (Bar Harbor, ME, USA). The double transgenic mice express a chimeric mouse/human amyloid precursor protein (Mo/HuAPP695swe) and a DeltaE9 mutant human presenilin 1 (PS1-dE9), both directed to CNS neurons. Both mutations are associated with the early onset of AD. The two transgenes were inserted at a single locus in Chromosome 9 between Arpp21 and Pdcd6ip. The “humanized” Mo/HuAPP695swe transgene allows the mice to secrete a human A-beta peptide. The included Swedish mutations (K595N/M596L) also elevate the amount of A-beta produced from the transgene by favoring processing through the beta-secretase pathway. Mice carrying this double transgene develop beta-amyloid deposits in the brain by 6–7 months of age. Hemizygous mice on the C57BL/6 background (N9B6) exhibit a high incidence of seizures (by 4.5 months of age, seizure incidence increases to 55%). Furthermore, these animals may display a slight alteration in their tail phenotype (e.g., kinked tail) that is believed to be due to the mixed genetic background of the strain and is not related to transgene expression. Mouse Strain Datasheet (2016)^1^.

Mice were 23–33 weeks old at the start of the experiment. Only male animals were used. All mice were maintained under a 12-h light/dark cycle (from 08:30 to 20:30) at constant temperature (22 ± 1°C) and humidity (55 ± 5%) with free access to food (rat chow pellets) and tap water. All experiments using live animals were performed in accordance with the protocols approved by the regional Hungarian Animal Health and Food Control Station and the University of Szeged (I-74-4/2011.MÁB.SZ). This study was carried out in strict accordance with EU Directive 2010/63/EU. Euthanasia of animals were performed under deep sodium pentobarbital (Nembutal) anesthesia, and all efforts were made to minimize suffering.

### Stress exposure

RS was applied based on our previous results (Santha et al., 2012, 2015). The animals were placed in well-ventilated, darkened, 50-ml centrifuge tubes for 6 h every morning (Freeman et al., 2007). Five groups were created from both mouse strains, every group consisted of 6 animals. The groups were the following: group 1/wt and 1/AD (*n* = 6) were controls; group 2/wt and 2/AD (*n* = 6) underwent 3 days of RS; group 3/wt and 3/AD underwent 7 days of RS; group 4/wt and 4/AD (*n* = 6) underwent 14 days of RS; group 5/wt and 5/AD (*n* = 6) underwent 21 days of RS. Control animals remained in their original boxes and were left undisturbed. The body weight of the animals was measured at the beginning of the experiment and after every RS treatment period (at 3, 7, 14, and 21 days).

### Tissue collection

RS exposed animals were anesthetized within 24 h from the last day of RS with a 50 mg/ml solution of sodium pentobarbital in sterile saline, administered IP at a dose of 70 mg/kg. Control animals (group 1) were euthanized with the 21-day RS animals (group 5). The animals under deep anesthesia were perfused transcardially with a 4°C, 0.9% normal saline solution, then the brain was removed, the hippocampus and prefrontal cortex were isolated from both the left and right hemispheres and the resulting samples were stored at −80°C until further use.

### Total DNA, RNA and protein isolation

Total cellular DNA, RNA and protein species were purified from the hippocampus and prefrontal cortex using the NucleoSpin® Triprep isolation kit (Macherey-Nagel GmbH & Co. KG, Düren, Germany), based on the manufacturer's instructions. 0.5 μl of RNase inhibitor 40 U/μl (Fermentas, Glen Burnie, ML, USA) was added to the eluted RNA. The resulting 40 μl eluted RNA,100 μl eluted genomic DNA (gDNA) and the 500 μl eluted protein samples' concentrations were measured with the Qubit 2.0 Fluorometer® (Life Technologies, Thermo Fisher Scientific Inc., Waltham, MA, USA) using the Qubit® High Sensitivity mRNA Assay Kit, High Sensitivity DNA Assay Kit and Protein Assay Kit, respectively. The samples were stored at −80°C until further use.

### RT-qPCR analysis of AdipoR1, AdipoR2 and LepR mRNA expression

The mRNA expression of AdipoR1, AdipoR2, and LepR were measured by a two-step real-time reverse transcription polymerase chain reaction (RT-qPCR) with the CFX96 Touch™ Real-Time PCR Detection System (Bio-Rad Laboratories Inc., Hercules, CA, USA).

The High Capacity cDNA Reverse Transcription kit (Life Technologies, Thermo Fisher Scientific Inc., Waltham, MA, USA) was used for reverse transcription, based on the manufacturer's instructions. From each sample 2 μg RNA was transcribed to cDNA. The total volume of each reaction was 30 μL. The three thermal cycle steps were the following: 25°C for 10 min, 37°C for 120 min, and 85°C for 5 s after which each sample was cooled down to 4°C. The transcribed cDNA samples were then diluted in 510 μL nuclease-free water and were stored at −20°C until further use.

At the qPCR step, SybrGreen-based detection was used for the measurement of AdipoR1 and R2, while TaqMan-based detection was applied for determining LepR. This was to prevent false results in the detection of LepR caused by transcript variants of highly similar sequence transcribed from the *LEPR* gene. For SybrGreen-based detection, qPCR was performed with a final volume of 20 μl, containing 9 μl of diluted cDNA, 0.5 μl of reverse primer, 0.5 μl of forward primer, and 10 μl of SYBR® Select Master Mix for CFX (Life Technologies, Thermo Fisher Scientific Inc., Waltham, MA, USA). The cDNA specific primers for AdipoR1, AdipoR2 and GAPDH mRNA were designed with the help of the NCBI Gene database and the NCBI Primer-BLAST tool. The cycle protocol provided by the manufacturer, repeated 40 times per run, was used for detection. At the end of each run, melt curve analysis was performed to ensure the quality of the acquired data. The analysis was carried out between 55°C and 93°C with a 0.2°C temperature increment per 10 s per step. The relative gene expression was normalized to GAPDH expression and the results were first analyzed with the ΔΔCq method (Livak and Schmittgen, 2001) and later with a gDNA-based quantification, detailed below. The sequence of primers are shown in Table 1.

**Table 1:** **List of primers used**.

**Gene**	**Forward 5′–3′**	**Reverse 5′–3′**	**Length of amplicons**
AdipoR1	CCTGTCCACCATCACAGGAA	GTGGGAAGACATCTGGCTGG	108
AdipoR2	AACTCTGACAGGATTTGGGGTC	CAATCGGTGTTTGGCTGGCT	133
GAPDH	TGTGTCCGTCGTGGATCTGA	TTGCTGTTGAAGTCGCAGGAG	150

For TaqMan-based detection, qPCR was performed with a final volume of 20 μl. The cycle protocol provided by the manufacturer was used for detection, which was repeated 40 times per run. The relative gene expression was normalized to GAPDH expression and the results were analyzed with the ΔΔCq method (Livak and Schmittgen, 2001).

### Quantification of RT-qPCR data

The RT-qPCR data for the AdipoR1 and R2 mRNA was quantified from a mouse gDNA standard curve according to Yun et al. (2006). From the previously isolated and measured mouse gDNA samples, five were randomly selected that were sheared by needle point shearing. After shearing, 5 quantities were produced from the cDNA samples with a threefold dilution ranging from 9 to 0.111 ng, respectively. The approximate AdipoR1 and R2 gene copy numbers of each quantity were calculated using the mass of the average haploid mouse genome and the adiponectin receptor genes' genomic copy number in mouse. All the gDNA dilutes of the different samples were amplified with the same protocol and primers used for the qPCR analysis described previously. From the resulting data a standard curve of C_t_ vs. copy number was generated.

### Elisa of AdipoR1 and AdipoR2

Mouse AdipoR1 specific antibody-coated, 96-well enzyme-linked immunosorbent assay (ELISA) plates and AdipoR2 specific antibody-coated, 96-well ELISA plates (Sanghai Sunred Biological Technology Co., Ltd., Shanghai, PRC) were used following the manufacturer's instructions. For the control and four stress-exposed groups of both mouse strains in our experiment, three protein samples were randomly selected from the six available samples. The total protein concentration loaded into each well was 20 μg/ml in a 40 μl volume. The final OD values were measured with the SpectraMax® Plus^384^ (Molecular Devices, LLC., Sunnyvale, CA, USA) absorbance microplate reader and sample protein concentration was calculated with the SoftMax® Pro Data Acquisition and Analysis Software (Molecular Devices, LLC., Sunnyvale, CA, USA).

### Statistical analysis

The stress-exposed groups were compared to their respective controls (per gene, per brain area and per mouse strain) by one-way analysis of variance (ANOVA) followed by Bonferroni and Tukey *post hoc* tests. The comparison between the same stress-treated, but different mouse strain, samples in every brain area for the studied genes was performed using Welch's two sample *t*-test. Results were considered to be significantly different at a probability level of *p* < 0.05 and lower (*p* < 0.01; *p* < 0.001). Data are presented as means plus standard error of means (SEM). For every statistical calculation we used the “R: A language and environment for statistical computing” (Version: 3.1.2; The R Foundation for Statistical Computing, Vienna, Austria) and the “R Studio: Integrated development environment for R” (Version: 0.98.1091, R Studio Co., Boston, MA, USA) statistical software. Plot.ly Software (Plotly Technologies Inc. Collaborative data science. Montréal, QC, 2016. https://plot.ly.) and its built-in algorithms were used to create the boxplot data presented in this article.

## Results

### AdipoR1 and AdipoR2 mRNA expression in the WT and APP/PS1 mice

Earlier studies focus mostly on the higher systemic concentration of adiponectin related to AD (Ishii and Iadecola, 2016), however the background of the adiponectin-AD connection remains unclear.

**Figure 1:**
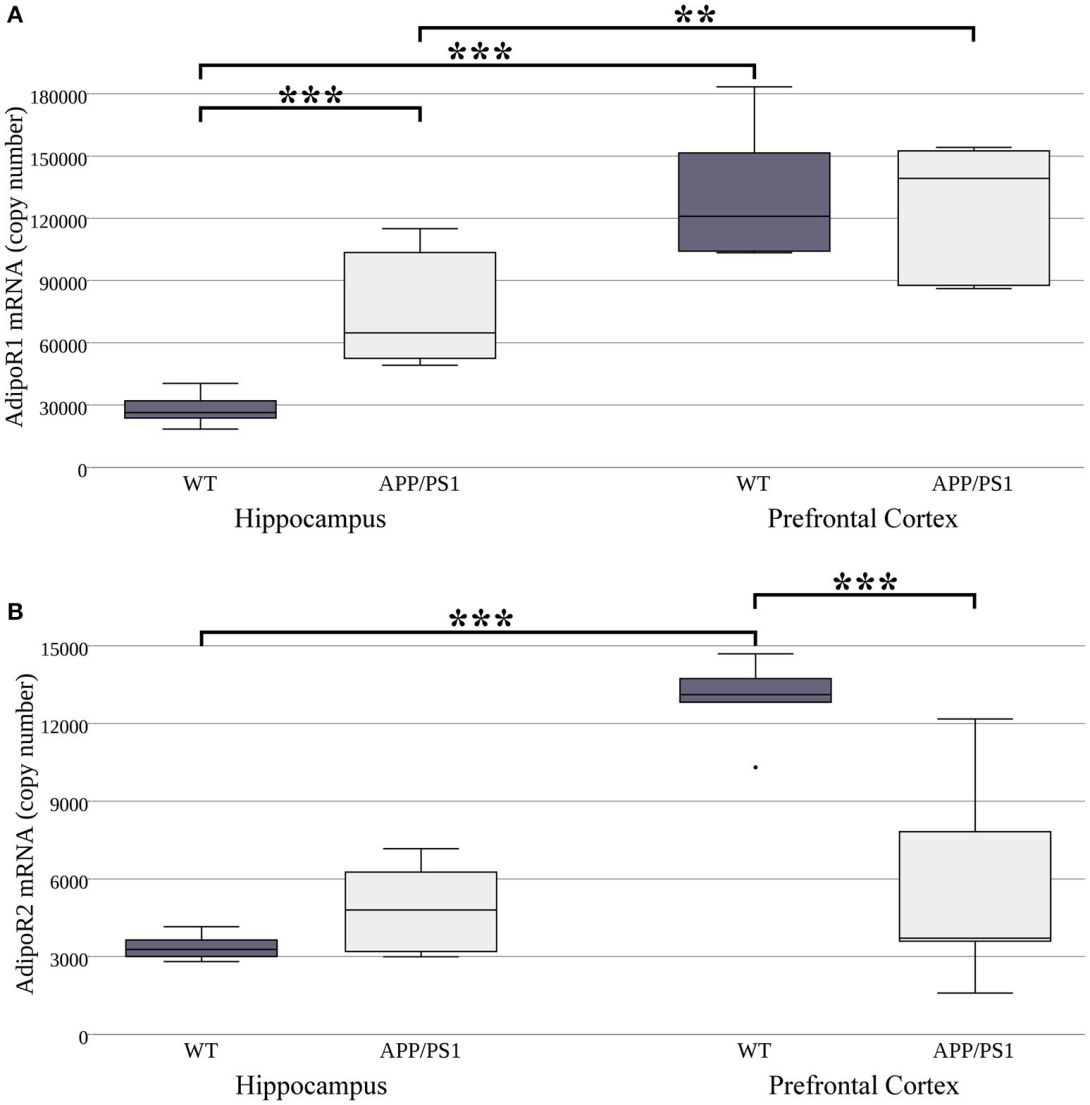
**AdipoR1 (A)** and AdipoR2 **(B)** mRNA expression in WT and AD mouse hippocampus and prefrontal cortex. The mouse strains were compared by Welch's two sample *t*-tests (per gene, per brain area), data are expressed as means + SEM. (*n* = 6), ^***^p < 0.001; ^**^p < 0.01.

For this reason, first we intended to compare the expression of AdipoR1 and AdipoR2 mRNA between WT and AD mice. Regarding the two examined brain regions, significantly higher AdipoR1 mRNA levels have been found in the prefrontal cortex compared to the hippocampus in both WT and APP/PS1 transgenic mice (Figure 1A). Additionally, AdipoR1 mRNA expression is significantly higher in the hippocampus of AD mice than in WT, while in the prefrontal cortex AdipoR1 mRNA did not differ between the two strains (Figure 1A). AdipoR2 hippocampal mRNA levels were equal in the WT and AD models, but in WT mice its expression (similarly to AdipoR1) was lower in the hippocampus compared to the prefrontal cortex (Figure 1B). Furthermore, AdipoR2 mRNA expression was much lower in the prefrontal cortex of AD animals compared to their WT counterparts (Figure 1B). Interestingly, mRNA expression of AdipoR1 was 8.3 and10 times higher compared to the expression of AdipoR2 in both the hippocampus and in the prefrontal cortex, respectively (Figure 1). In APP/PS1 mice, since only the AdipoR1 mRNA levels elevated and AdipoR2 mRNA levels did not, the difference in the expression between the two receptors further increased up to 15.5 and 23 times in the hippocampus and prefrontal cortex, respectively. Taken together, these data show that there are indeed differences in adiponectin receptor mRNA expression between the WT and APP/PS1 transgenic mice and even between the two examined brain areas in each strain.

### AdipoR1 and AdipoR2 proteins in the WT and the APP/PS1 mice

**Figure 2:**
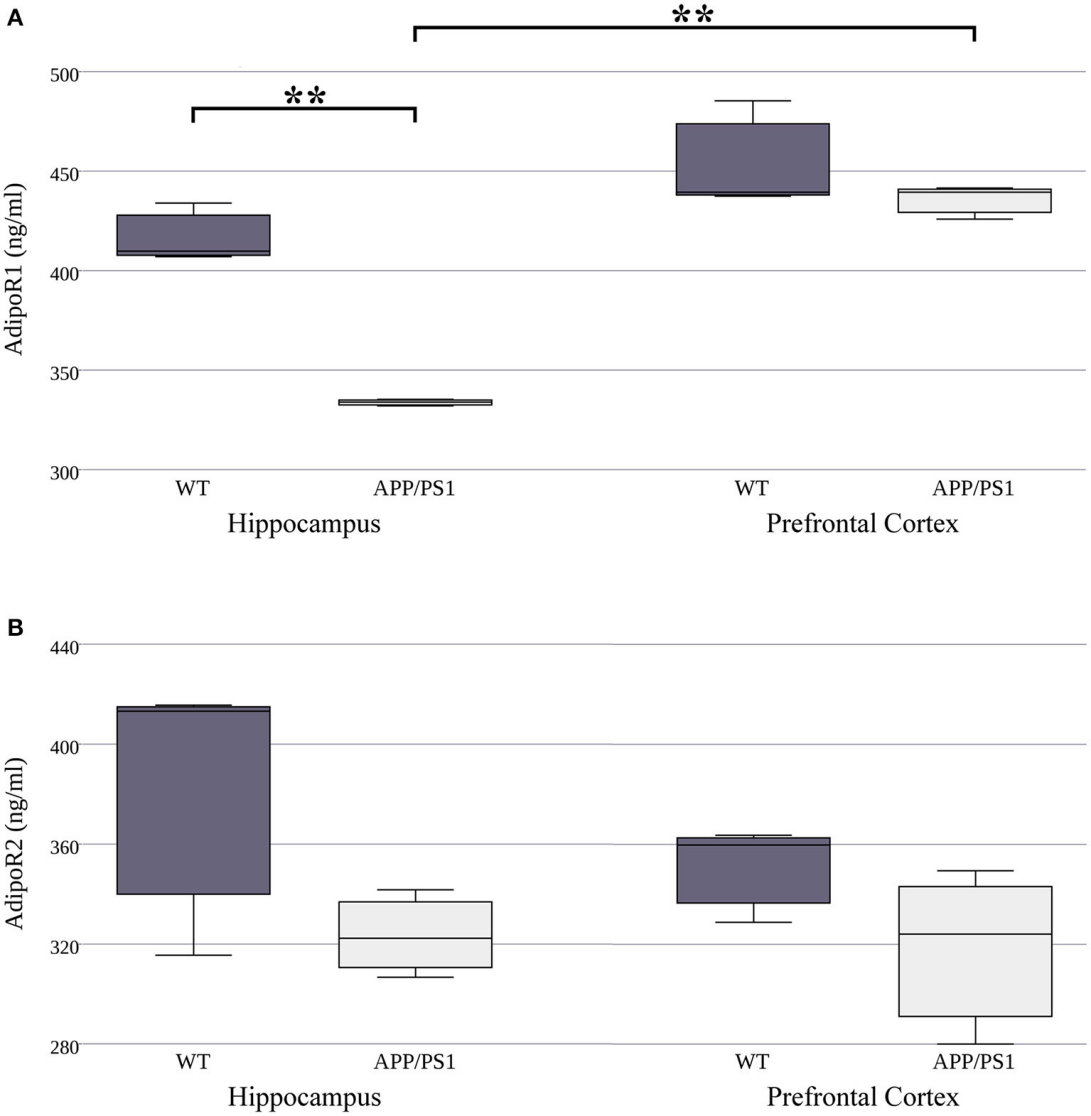
**AdipoR1 (A)** and AdipoR2 **(B)** protein expression in WT and AD mouse hippocampus and prefrontal cortex. The mouse strains were compared by Welch's two sample *t*-tests (per gene, per brain area), data are expressed as means + SEM. (*n* = 3), ^**^p < 0.01.

Our next goal was to investigate whether there are similar differences in adiponectin receptor protein concentrations to what was observed in the mRNA levels. We used ELISA kits to measure the exact protein concentration of AdipoR1 and AdipoR2 in both strains and both brain areas. Lower hippocampal AdipoR1 protein expression was detected in the APP/PS1 transgenic mice compared to the WT ones (Figure 2A, Supplementary Table 2). Furthermore, in the WT strain there were no differences in protein level of either receptor between the two regions. However, in AD mice, AdipoR1 receptor levels were lower in the hippocampus than in the prefrontal cortex (Figure 2A). We found no significant differences in AdipoR2 hippocampal and prefrontal cortical protein expression between WT and AD animals (Figure 2B). In summary, these data demonstrate that protein levels differ greatly from mRNA expression for the two adiponectin receptors. The 8.3–10 fold difference between AdipoR1 and AdipoR2 which we observed at the mRNA level was almost completely abolished at the protein level. These results may suggest strong, genetically determined pre- and/or post-translational regulation of these receptors.

### The effect of restraint stress on AdipoR1 and AdipoR2 mRNA expression

**Figure 3:**
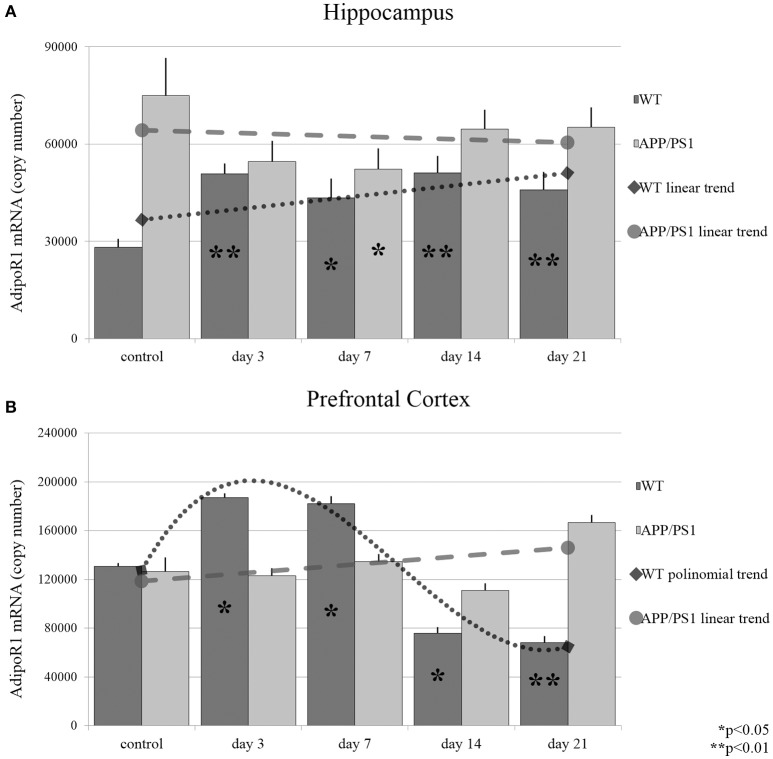
**Effects of restraint stress on the expression of AdipoR1 mRNA in the hippocampus (A)** and prefrontal cortex **(B)** of the wild type and APP/PS1 transgenic mice. Stress-exposed groups were compared to their respective controls (per gene, per brain area, and per mouse strain) by one-way analysis of variance (ANOVA) followed by Bonferroni and Tukey *post hoc* tests. Data are expressed as means + SEM (*n* = 6), ^*^p < 0.05; ^**^p < 0.01. The gray lines represent linearized (**A,B**, APP/PS1 strain) and polynomial (**B**, WT strain) trends of change in the two strains.

Long-term mild common stressors may influence the risk of AD development during an individual's lifetime. It has been shown, that adiponectin expression is sensitive to environmental stress. To gain further information related to stress-induced changes, we wanted to investigate whether acute or chronic stresses can influence the expression of AdipoR1 and AdipoR2 mRNA levels. As shown in Figure 3, both acute (day 3) and chronic (day 7, 14, and 21) RS caused significant, almost twofold, elevations in AdipoR1 mRNA expression in the hippocampus of WT mice, while in APP/PS1 mice only a smaller decrease could be observed after 7 days of stress treatment (Figure 3A). In the prefrontal cortex, the mRNA expression of AdipoR1 followed a biphasic course, it increased in the cases of the shorter RS (3 day and day 7), while a sharp drop could be observed after a longer period of RS (day 14 and day 21) (Figure 3B). These data demonstrate that even really short term (3 day) stress can induce an elevation in AdipoR1 mRNA production in both examined brain areas of WT mice. This is consistent with a previous report, stating that repeated exposure to stresses causes enhanced gene expression of adiponectin in gonadal white adipose tissue. In AD mice, RS did not induce any change in the expression of AdipoR1 mRNA either in the hippocampus (Figure 3A) or the prefrontal cortex (Figure 3B), except a transient decrease in hippocampal AdipoR1 mRNA in the 7th day (Figure 3A).

**Figure 4:**
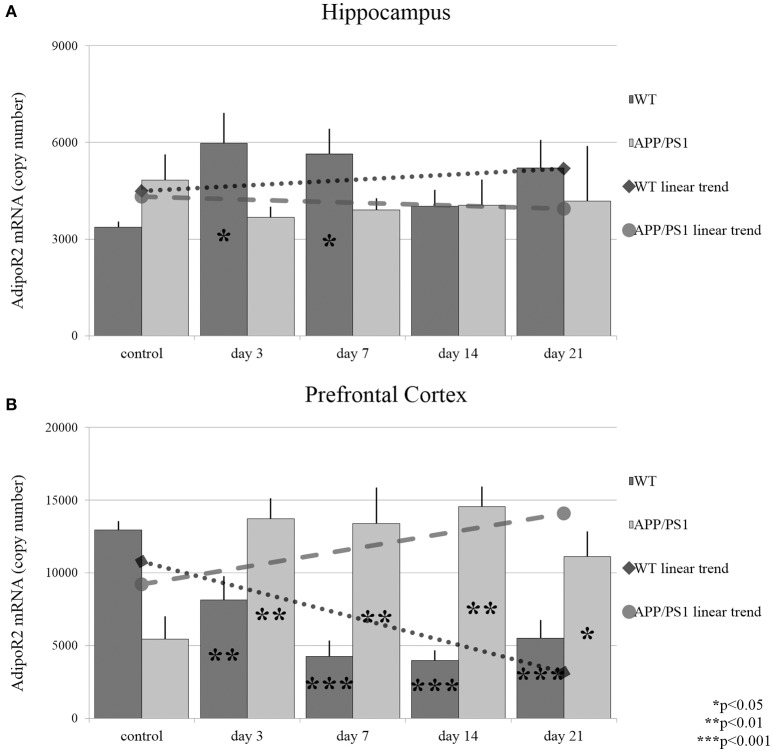
**Effects of restraint stress on the expression of AdipoR2 mRNA in the hippocampus (A)** and prefrontal cortex **(B)** of the wild type and APP/PS1 transgenic mice. Stress-exposed groups were compared to their respective controls (per gene, per brain area and per mouse strain) by one-way analysis of variance (ANOVA) followed by Bonferroni and Tukey *post hoc* tests. Data are expressed as means + SEM (*n* = 6), ^*^p < 0.05; ^**^p < 0.01, and ^***^p < 0.001. The gray lines represent linearized trends of change in the two strains.

AdipoR2 mRNA reacted to stress in the hippocampus largely in a similar manner to what was observed for AdipoR1 in WT mice: The expression of AdipoR2 mRNA increased significantly only on days 3 and 7 (Figure 4A). In AD mice, hippocampal AdipoR2 mRNA levels remained unchanged at every time point during RS. In the prefrontal cortex of WT mice, the AdipoR2 mRNA expression showed a steady decrease during RS, which reached its lowest levels by day 7 and 14 (Figure 4B). In contrast, AdipoR2 mRNA localized in the prefrontal cortex of AD animals was found to be strongly elevated by days 3, 7, and 14 and moderately by day 21 (Figure 4B). Thus, our results showed a completely different pattern for AdipoR2's prefrontal cortical expression in our two strains with the receptor's expression decreasing in the WT and increasing in the transgenic animals. Exact points of adiponectin receptor mRNA expression data are summarized in the Supplementary Table 1.

### The effect of restraint stress on AdipoR1 and AdipoR2 protein expression

**Figure 5:**
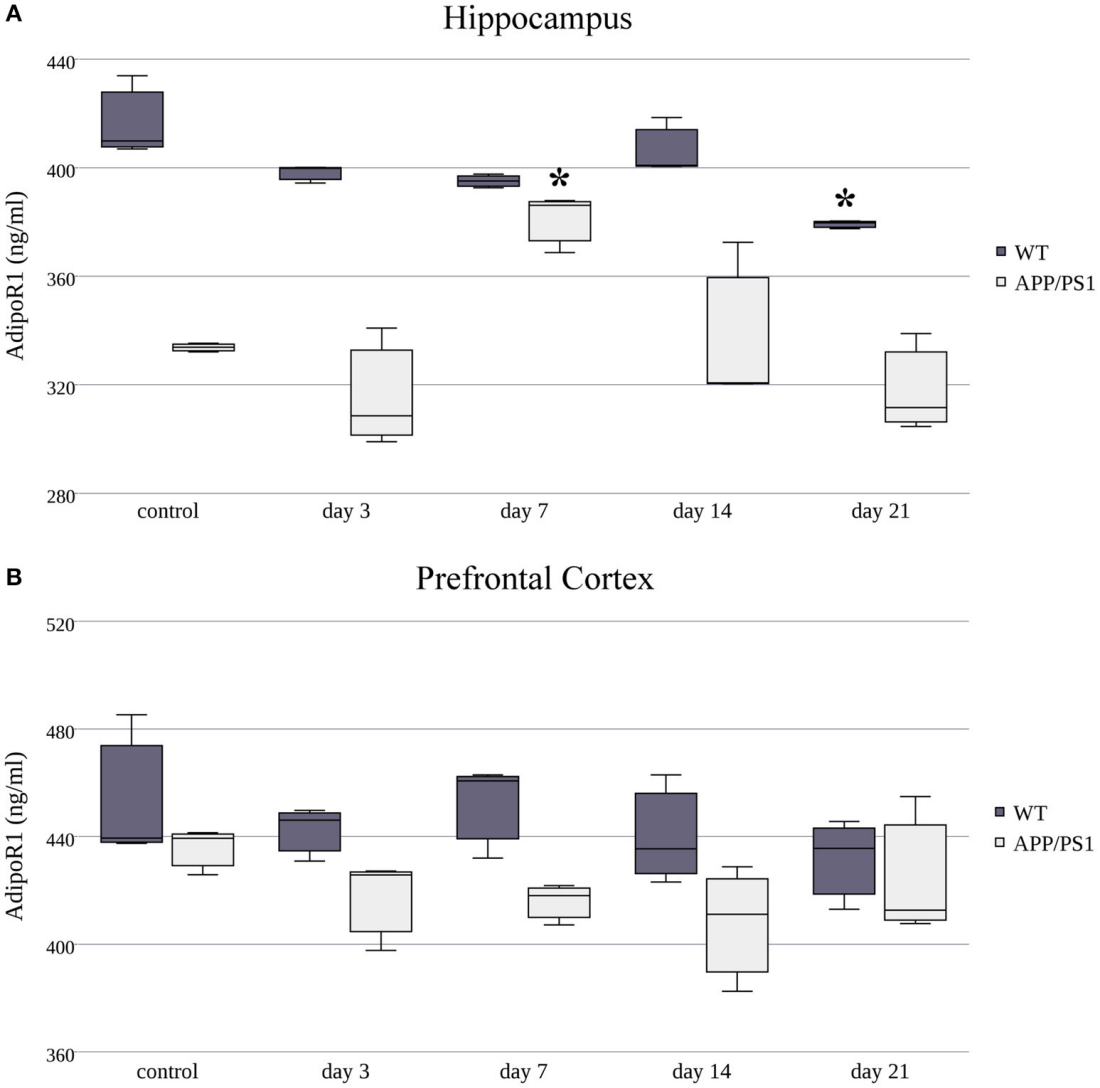
**Effects of restraint stress on the expression of AdipoR1 protein in the hippocampus (A)** and prefrontal cortex **(B)** of the wild type and APP/PS1 transgenic mice. Stress-exposed groups were compared to their respective controls (per protein, per brain area, and per mouse strain) by one-way analysis of variance (ANOVA) followed by Bonferroni and Tukey *post hoc* tests. Data are expressed as means + SEM (*n* = 3), ^*^p < 0.05.

Next, we measured AdipoR1 and AdipoR2 protein concentrations using ELISA kits on three randomly selected samples in both strains at each time point of RS. The protein expression of AdipoR1 showed quite the opposite to what we saw at the mRNA level. Despite the fast and constant elevation in AdipoR1 mRNA in the hippocampus of WT mice, a significant decrease in AdipoR1 protein levels was induced by chronic, 21-day stress (Figure 5A). Hippocampal AdipoR1 expression in AD mice showed an elevation on day 7 (Figure 5A), in contrast to mRNA expression, which was lower in the 7-day group compared to the control. In the prefrontal cortex, RS did not cause any significant changes in AdipoR1 protein levels in either WT or AD mice (Figure 5B), meaning that the biphasic change we observed in the WT AdipoR1 mRNA concentration did not translate to the protein level.

**Figure 6:**
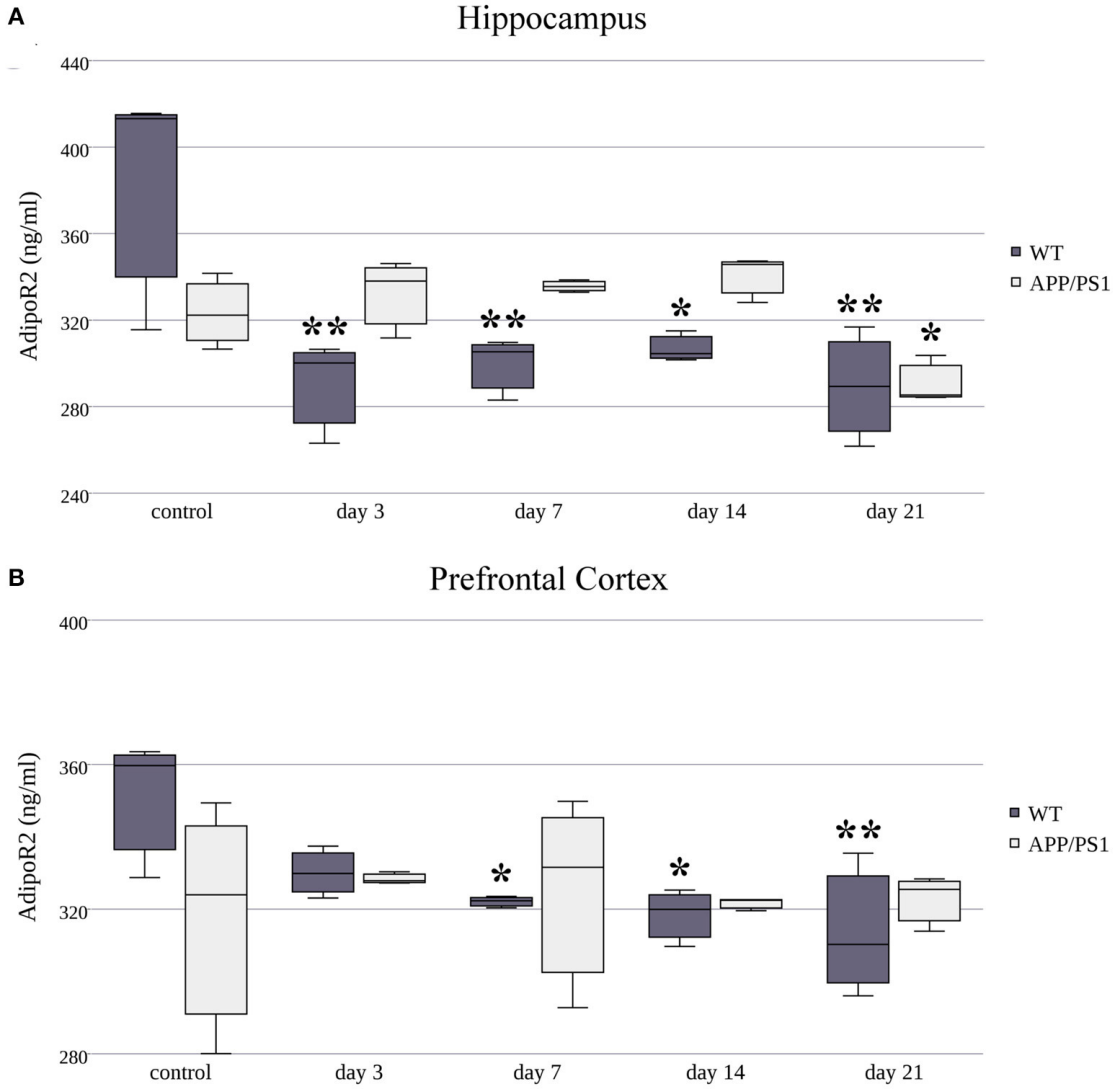
**Effects of restraint stress on the expression of AdipoR2 protein in the hippocampus (A)** and prefrontal cortex **(B)** of the wild type and APP/PS1 transgenic mice. Stress-exposed groups were compared to their respective controls (per protein, per brain area, and per mouse strain) by one-way analysis of variance (ANOVA) followed by Bonferroni and Tukey *post hoc* tests. Data are expressed as means + SEM (*n* = 3), ^*^p < 0.05; ^**^p < 0.01.

As in AdipoR1, AdipoR2's protein levels were decreased in the hippocampus of WT mice in every stress-exposed group (day 3, 7, 14, or 21) (Figure 6A), despite the increased production of its mRNA caused by short term (3 and 7 day) stress. As for the AD animals, only the longer, chronic (day 21) RS induced a mild, but significant, decrease in AdipoR2 protein levels in the hippocampus compared to the control group. In the prefrontal cortex of WT mice, AdipoR2 levels showed the same tendency as in the hippocampus, the reduction was significant on the 7th, 14th, and 21st days (Figure 6B). This AdipoR2 protein expression pattern was the only dataset where the protein levels matched the mRNA levels. Furthermore, in stress-exposed AD animals, the prefrontal cortical levels of AdipoR2 did not differ from those of the control group (Figure 6B). Exact points of protein adiponectin receptor expression data are summarized in the Supplementary Table 2.

### The effect of restraint stress on LepR mRNA expression in WT and APP/PS1 animals

It can be suggested, that the decreased level of LepR may be a possible cause of leptin insensitivity, which has been described in AD. Therefore, we used TaqMan based PCR chemistry to precisely measure the LEPR long isoform mRNA in our samples, which is solely responsible for coding LepR.

**Figure 7:**
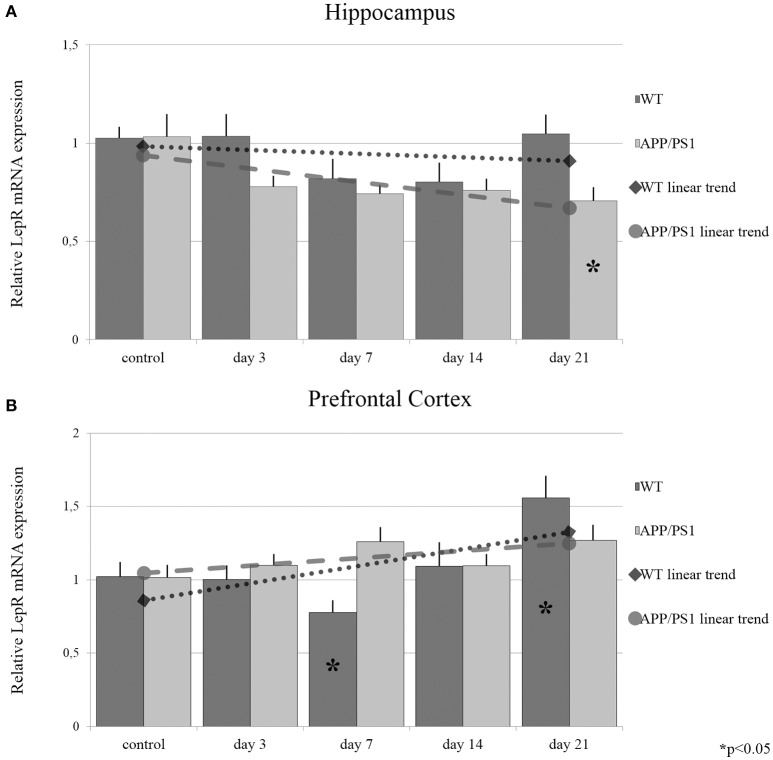
**Effects of restraint stress on the expression of LepR mRNA in the hippocampus (A)** and prefrontal cortex **(B)** of the wild type and APP/PS1 transgenic mice. Stress-exposed groups were compared to their respective controls (per protein, per brain area, and per mouse strain) by one-way analysis of variance (ANOVA) followed by Bonferroni and Tukey *post hoc* tests. Data are expressed as means + SEM. (*n* = 6), ^*^p < 0.05. The gray lines represent linearized trends of change in the two strains.

The resulting data indicated that RS did not induce any consistent changes in the hippocampal LepR mRNA expression in WT mice (Figure 7A). However, in the prefrontal cortical area, chronic RS resulted in a significant decrease in LepR mRNA expression by day 7. Then LepR mRNA returned to the control group's level again by day 14, and finally elevated significantly by day 21 (Figure 7B). In APP/PS1 transgenic mice, only the longer, 21-day RS caused significant LepR mRNA reduction in the hippocampus (Figure 7A). In conclusion, for LepR we only identified a few significant differences in our stress-treated groups, implying that LepR's expression is not affected prominently by the APP/PS1 transgenes or by stress. Furthermore, these data are consistent with the results of Balland and colleagues, who found that LepR regulation and availability is not an element in the leptin resistance mechanisms. Exact points of LepR mRNA expression data are summarized in Supplementary Table 1.

## Discussion

The significant contribution of adiponectin and leptin in the pathomechanism of AD has been highly researched in the past few years (Tezapsidis et al., 2009; van Himbergen et al., 2012; Pedros et al., 2015). However, the exact role of these molecules in the disease is still very elusive, thanks to their complex interactions with the CNS. Adiponectin and leptin may not only be associated with AD via their changed serum concentrations, but also through other means. The possible target points regarding the CNS may be the signaling pathways, more precisely the receptors of these adipokines (Waragai et al., 2016). Taking this into consideration, the current study shows that bearing the APP/PS1 transgenes associated with AD induces altered expression of AdipoR1 and AdipoR2 in the hippocampus and the prefrontal cortex. The present paper also demonstrates that chronic stress, a potential AD inducer, may influence the levels of both adiponectin receptors in the aforementioned brain areas of wild type mice. Furthermore, the same receptors responded less or not at all to identical chronic RS in the APP(swe)/PS1(e9d)1 transgene murine model of AD. The differences in stress response were stronger at the protein level. Considering LepR mRNA in the same strains and brain areas, we only detected slight modifications compared to the adiponectin receptors.

The presence of AdipoR1 and AdipoR2 in several brain areas, including the frontal cortex and the hippocampus, has been shown previously (Qiu et al., 2011). However, information on the AD-related changes of AdipoR1 and AdipoR2 is still very limited. Therefore, in this study, the expression of adiponectin receptors mRNA and proteins were quantified for the first time in WT and APP/PS1 transgenic mice. In contrast with the increased transcription of the hippocampal AdipoR1, the presence of the two AD-related transgenes decreased the AdipoR1 protein levels. These results assume a downregulated signalization of adiponectin in AD and support earlier findings (Waragai et al., 2016). In this cross-sectional study, pathologically changed adiponectin-adiponectin receptor signaling was suggested based on the higher serum and lower CSF adiponectin levels in AD patients.

Based on the fact that chronic stress has considerable effects on cognitive decline and memory loss, a possible link between psychological stress and AD development has been suggested (Khalsa, 2015). Our results show that stress stimuli can downregulate AdipoR1 and AdipoR2 transcription and translation in the hippocampal and prefrontal cortical areas of wild type mice. The question arises as to what this stress-induced modification of adipokine receptors really means for the pathomechanism of AD. Adiponectin has been reported to have anti-inflammatory, anti-atherogenic, anti-diabetogenic and neuroprotective effects (Berg et al., 2002; Qiu et al., 2011; Ohashi et al., 2012). According to our observations, because of the decreased availability of AdipoR1 and AdipoR2, the beneficial properties of adiponectin may diminish during stress; thus adiponectin may fail to exert its protective effects against neuronal cell death in the hippocampus and in the prefrontal cortex. It can be concluded that chronic stress, due to the decreased availability of adiponectin receptors, may contribute to the loss of the favorable effects of adiponectin and, consequently, may accelerate the progression of AD. Additionally, we could not demonstrate similar modulating effects of stress in the transgenic murine model of AD, since the mRNA and protein levels of adiponectin receptors were not reduced. These unexpected results suggest that the availability of adiponectin receptors can be stabilized even in cases where genetic and environmental risk factors for AD co-occur. Recently, Han et al. (2016) demonstrated that insulin receptor levels are decreased in the hippocampus of AD mice and can further decline after stress exposure, concluding that people with harmful genetic mutations are more likely to be vulnerable to stress. Further investigation is necessary to explore the mechanism to explain the difference between the stress-induced changes of adiponectin and insulin receptors.

RS affected adiponectin receptor protein levels less remarkably than their mRNA, which is not an unexpected finding since the proteome is much more stable than the mRNA levels. It is well known, that expression of a given mRNA and protein do not always match completely, mainly due to post-transciptional and post-translational control mechanisms. As post-transcriptional regulators of gene expression, microRNAs may be partly responsible for the disparate expression of adiponectin receptors at the protein and RNA levels. A recent bioinformatics analysis showed that adiponectin signaling is regulated by microRNAs: miR-221 inhibits AdipoR1 expression, which suggests that miR-221 regulates AdipoR1 signaling in different pathological processes (Chen et al., 2015). On the other hand, AdipoR2 has been identified as a direct target of miR-218 (Du et al., 2015). In our experiments, the expression of AdipoR2 in the prefrontal cortex of AD mice increased significantly at all time-points of RS, however these changes could not be measured at the protein level. One potential explanation of our observations may be the role of miR-218 in this signaling process, which nevertheless needs further clarification.

The most well characterized adipokine for its role in AD is leptin (Folch et al., 2012). Population-based studies indicate that decreased leptin levels are associated with cognitive impairment (Perez-Gonzalez et al., 2011; Furiya et al., 2013), while higher leptin levels were associated with a lower risk of dementia (Lieb et al., 2009; Warren et al., 2012). However, leptin is also involved in the stress response and stress-related disorders, including depression and anxiety (Haleem, 2014). Both acute and chronic stress modify the circulating levels of leptin depending on the duration of the stress and the stress type itself (Haleem, 2014). Investigation of the effect of RS on LepR's mRNA expression in both WT and AD mice showed that LepR is less sensitive to RS than the adiponectin receptors, since only long-term, chronic RS decreased the transcription of LepR in the hippocampus of transgenic mice. This is consistent with previous data obtained from the Alzheimer's Disease Neuroimaging Initiative (ADNI) cohort study, which showed decreased LepR immunoreactivity in brains from individuals suffering severe AD, and similarly the LepR mRNA was also reduced only in the old AD transgenic mice (Maioli et al., 2015). Thus, we propose that APP/PS1 transgenes and RS do not affect LepR's transcription as much as they affect the adiponectin receptors' transcription. The mechanism of leptin resistance also seems to be independent from LepR regulation, which also coincides with our results (Balland and Cowley, 2015).

Our experiment had several limitations which must be considered during the evaluation of results. ELISA assays were performed only for AdipoR1 and AdipoR2 and we only used three randomly selected protein samples from the six available for each group to represent one group from the viewpoint of stress exposure. Thus, a major limitation in the interpretation of our results is the low number of samples. A study with a higher sample size may strengthen some of the observations made in this paper. Furthermore, we did not measure changes in serum cortisol throughout the experiment, which is one of the best indicators of psychological stress. The reason behind this decision was twofold; on the one hand, the stress paradigm we used is well-documented in the scientific literature to trigger molecular stress responses in murine models, including the increase in corticosterone and adrenocorticotropic hormone secretion (Buynitsky and Mostofsky, 2009). On the other hand, we wanted to monitor the effect of stress less invasively with weight measurements to avoid potential alterations in the resulting data due to the stress of blood sampling.

## Conclusion

In summary, we were the first to quantitatively investigate adiponectin receptors concentrations in AD-associated areas of the brain, namely the hippocampus and the prefrontal cortex. Based on our results, the expression of adiponectin receptors AdipoR1 and AdipoR2 are affected by genes APP and PS1 in a region-specific manner in the brain. These receptor molecules of adipokine frequently associated with AD are also influenced by chronic stress in a possibly neurodegeneration-promoting manner in the hippocampus and prefrontal cortex of C57BL/6J WT mice. We also demonstrated that in a model of fully developed AD, an APP(swe)/PSEN(e9d)1 transgene mouse strain, these receptors respond less plastically or not at all to the same chronic stress treatment. Furthermore, based on our observations, LepR plays an essential role only in the late phases of AD. These results also imply that variation in LepR expression is not an element of leptin resistance mechanisms.

## Author contributions

Conceptualization, ZV, MP, and JK; Methodology, ZV and MP; Investigation, ZV, EF, ZO, and EI; Formal Analysis, ZV; Writing – Original Draft, ZV and MP; Writing – Review and Editing, JK and ZD; Visualization, ZV; Funding Acquisition, MP and JK; Resources MS and JK; Supervision MP, JK, and ZD.

## Funding

This work was supported by the Hungarian Research and Technology Innovation Fund through the Hungarian Brain Research Program (KTIA_13_NAP-A-II/16).

### Conflict of interest statement

The authors declare that the research was conducted in the absence of any commercial or financial relationships that could be construed as a potential conflict of interest.

## Footnotes

^1^B6C3-Tg(APPswe,PSEN1dE9)85Dbo/Mmjax Mouse Strain Datasheet (2016).2017.
