# Supplementary material for: Adiponectin Receptors Are Less Sensitive to Stress in a Transgenic Mouse Model of Alzheimer's Disease
Source: Front Neurosci. 2017 Apr 11;11:199. doi: 10.3389/fnins.2017.00199 (PMC5386987; doi:10.3389/fnins.2017.00199)
Supplement: Supplementary file 1 [file Table1.docx]

Supplementary Table 1. Effects of restraint stress on the expression of AdipoR1, AdipoR2 and leptin receptor (LepR) mRNA in the hippocampus and prefrontal cortex of the wild type and APP/PS1 transgenic mice. Stress-exposed groups were compared to their respective controls (per gene, per brain area and per mouse strain) by one-way analysis of variance (ANOVA) followed by Bonferroni and Tukey post hoc tests. Data are expressed as means + SEM. (n=6/group), *p < 0.05; **p < 0.01; ***p < 0.001.

|  |  | **mRNA** | | | | | | | | | | |
| --- | --- | --- | --- | --- | --- | --- | --- | --- | --- | --- | --- | --- |
|  |  | **Hippocampus** | | | | |  | **Prefrontal Cortex** | | | | |
| **Strain** | **Restraint Stress** | **AdipoR1**† |  | **AdipoR2**† |  | **LepR**# |  | **AdipoR1**† |  | **AdipoR2**† |  | **LepR**# |
| ***WT*** | *Control* | 28105 ± 2694 |  | 3364 ± 174 |  | 1.03 ± 0.06 |  | 130714 ± 12754 |  | 12963 ± 601 |  | 1.02 ± 0.1 |
|  | *3 Day* | 50859 ± 3214** |  | 5973 ± 950* |  | 1.04 ± 0.11 |  | 187163 ± 18902* |  | 8135 ± 1629** |  | 1 ± 0.1 |
|  | *7 Day* | 43364 ± 5987* |  | 5646 ± 784* |  | 0.82 ± 0.1 |  | 182083 ± 16480* |  | 4246 ± 1093*** |  | 0.78 ± 0.08* |
|  | *14 Day* | 51129 ± 5146** |  | 4027 ± 507 |  | 0.8 ± 0.1 |  | 75839 ± 14101* |  | 3971 ± 702*** |  | 1.09 ± 0.16 |
|  | *21 Day* | 45847 ± 5584** |  | 5212 ± 878 |  | 1.05 ± 0.1 |  | 68056 ± 11265** |  | 5511 ± 1256*** |  | 1.56 ± 0.15* |
| ***APP/SWE*** | *Control* | 74966 ± 11542 |  | 4837 ± 793 |  | 1.03 ± 0.12 |  | 126495 ± 13054 |  | 5433 ± 1584 |  | 1.01 ± 0.09 |
|  | *3 Day* | 54638 ± 6314 |  | 3672 ± 334 |  | 0.78 ± 0.06 |  | 122761 ± 10213 |  | 13734 ± 1392** |  | 1.01 ± 0.08 |
|  | *7 Day* | 52336 ± 6316* |  | 3905 ± 359 |  | 0.74 ± 0.05 |  | 134577 ± 12658 |  | 13409 ± 2458** |  | 1.26 ± 0.1 |
|  | *14 Day* | 64664 ± 5944 |  | 4051 ± 803 |  | 0.76 ± 0.06 |  | 111000 ± 10146 |  | 14575 ± 1366** |  | 1.09 ± 0.1 |
|  | *21 Day* | 65150 ± 6114 |  | 4187 ± 1709 |  | 0.71 ± 0.07* |  | 166615 ± 21994 |  | 11119 ± 1741* |  | 1.27 ± 0.11 |
| † *mean of mRNA copy number + SEM (quantified mRNA)*  # *mean of relative mRNA expression + SEM (∆∆Cq method)* | | | | | | | | | | | | |
