# Supplementary material for: Adiponectin Receptors Are Less Sensitive to Stress in a Transgenic Mouse Model of Alzheimer's Disease
Source: Front Neurosci. 2017 Apr 11;11:199. doi: 10.3389/fnins.2017.00199 (PMC5386987; doi:10.3389/fnins.2017.00199)
Supplement: Supplementary file 2 [file Table2.docx]

Supplementary Table 2. Effects of restraint stress on the expression of AdipoR1 and AdipoR2 protein in the hippocampus and prefrontal cortex of the wild type and APP/PS1 transgenic mice. Stress-exposed groups were compared to their respective controls (per protein, per brain area and per mouse strain) by one-way analysis of variance (ANOVA) followed by Bonferroni and Tukey post hoc tests. Data are expressed as means + SEM. (n=3/group), *p < 0.05; **p < 0.01; ***p < 0.001.

|  |  | **Protein (mean protein concentration in ng/ml + SEM)** | | | | | | |
| --- | --- | --- | --- | --- | --- | --- | --- | --- |
|  |  | **Hippocampus** | | |  | **Prefrontal Cortex** | | |
| **Strain** | **Restraint Stress** | **AdipoR1** |  | **AdipoR2** |  | **AdipoR1** |  | **AdipoR2** |
| ***WT*** | *Control* | 424.9 ± 16.2 |  | 381.4 ± 33 |  | 454 ± 15.6 |  | 350.7 ± 11 |
|  | *3 Day* | 396.3 ± 10.5 |  | 290 ± 13.6** |  | 442.2 ± 5.8 |  | 330.1 ± 4.1 |
|  | *7 Day* | 396.2 ± 4.3 |  | 299.4 ± 8.3** |  | 451.9 ± 9.9 |  | 322.1 ± 0.9* |
|  | *14 Day* | 416.5 ± 13.9 |  | 307.1 ± 4.1* |  | 440.5 ± 11.8 |  | 318.3 ± 7.9* |
|  | *21 Day* | 365.6 ± 17.9* |  | 289.3 ± 15.9** |  | 431.4 ± 9.7 |  | 322.9 ± 11.5** |
| ***APP/SWE*** | *Control* | 335.1 ± 7.1 |  | 323.6 ± 10.1 |  | 435.5 ± 4.9 |  | 317.8 ± 20.3 |
|  | *3 Day* | 323.5 ± 15.9 |  | 332 ± 10.4 |  | 416.9 ± 9.6 |  | 328.5 ± 0.9 |
|  | *7 Day* | 376.2 ± 8.4* |  | 335.7 ± 1.6 |  | 415.7 ± 4.4 |  | 324.8 ± 16.8 |
|  | *14 Day* | 336.6 ± 15.5 |  | 340.4 ± 6.1 |  | 407.4 ± 13.5 |  | 321.6 ± 1 |
|  | *21 Day* | 323.1 ± 18 |  | 291 ± 6.3* |  | 425 ± 15 |  | 322.6 ± 4.4 |
|  | | | | | | | | |
